# Regional hippocampal atrophy reflects memory impairment in patients with early relapsing remitting multiple sclerosis

**DOI:** 10.1007/s00415-024-12290-8

**Published:** 2024-05-14

**Authors:** Rosa Cortese, Marco Battaglini, Maria Laura Stromillo, Ludovico Luchetti, Matteo Leoncini, Giordano Gentile, Daniele Gasparini, Domenico Plantone, Manuela Altieri, Alessandro D’Ambrosio, Antonio Gallo, Costanza Giannì, Claudia Piervincenzi, Patrizia Pantano, Elisabetta Pagani, Paola Valsasina, Paolo Preziosa, Nicolo’ Tedone, Maria Assunta Rocca, Massimo Filippi, Nicola De Stefano, INNI Network, Alvino Bisecco, Fabrizio Esposito, Alessandro De Rosa, Serena Ruggieri, Silvia Tommasin, Nikolaos Petsas, Loredana Storelli, Stefania Sala

**Affiliations:** 1 Department of Medicine, Surgery and Neuroscience, University of Siena, Viale Bracci 2, 53100 Siena, Italy; 2 SIENA Imaging SRL, 53100 Siena, Italy; 3 Department of Advanced Medical and Surgical Sciences, University of Campania “Luigi Vanvitelli”, Piazza Luigi Miraglia, 2, 80138 Naples, Italy; 4 Department of Human Neurosciences, Sapienza University of Rome, Rome, Italy; 5 IRCCS Neuromed, Pozzilli, IS Italy; 6 Neuroimaging Research Unit, Division of Neuroscience, IRCCS San Raffaele Scientific Institute, Milan, Italy; 7 Neurology Unit, IRCCS San Raffaele Scientific Institute, Milan, Italy; 8 Neurorehabilitation Unit, IRCCS San Raffaele Scientific Institute, Milan, Italy; 9 Neurophysiology Service, IRCCS San Raffaele Scientific Institute, Milan, Italy; 10 Vita-Salute San Raffaele University, Milan, Italy

**Keywords:** Multiple sclerosis, Hippocampal atrophy, Memory impairment, MRI

## Abstract

**Background:**

Research work has shown that hippocampal subfields are atrophic to varying extents in multiple sclerosis (MS) patients. However, studies examining the functional implications of subfield-specific hippocampal damage in early MS are limited. We aim to gain insights into the relationship between hippocampal atrophy and memory function by investigating the correlation between global and regional hippocampal atrophy and memory performance in early MS patients.

**Methods:**

From the Italian Neuroimaging Network Initiative (INNI) dataset, we selected 3D-T1-weighted brain MRIs of 219 early relapsing remitting (RR)MS and 246 healthy controls (HC) to identify hippocampal atrophic areas. At the time of MRI, patients underwent Selective-Reminding-Test (SRT) and Spatial-Recall-Test (SPART) and were classified as mildly (MMI-MS: n.110) or severely (SMI-MS: n:109) memory impaired, according to recently proposed cognitive phenotypes.

**Results:**

Early RRMS showed lower hippocampal volumes compared to HC (p < 0.001), while these did not differ between MMI-MS and SMI-MS. In MMI-MS, lower hippocampal volumes correlated with worse memory tests (r = 0.23–0.37, p ≤ 0.01). Atrophic voxels were diffuse in the hippocampus but more prevalent in cornu ammonis (CA, 79%) than in tail (21%). In MMI-MS, decreased subfield volumes correlated with decreases in memory, particularly in the right CA1 (SRT-recall: r = 0.38; SPART: r = 0.34, p < 0.01). No correlations were found in the SMI-MS group.

**Conclusion:**

Hippocampal atrophy spreads from CA to tail from early disease stages. Subfield hippocampal atrophy is associated with memory impairment in MMI-MS, while this correlation is lost in SMI-MS. This plays in favor of a limited capacity for an adaptive functional reorganization of the hippocampi in MS patients.

**Supplementary Information:**

The online version contains supplementary material available at 10.1007/s00415-024-12290-8.

## Introduction

Multiple sclerosis (MS) is a chronic immune-mediated inflammatory disease of the central nervous system, characterized by the involvement of white matter (WM) and gray matter (GM) since the early stages [1]. The hippocampus, a complex structure in the medial temporal lobe responsible for learning and episodic memory, has consistently shown abnormalities in MS [2]. Memory impairment is common in MS and can be observed early and across disease phenotypes [3, 4]. MS patients often experience difficulties in episodic memory, visual-spatial ability, and short-term working memory, while semantic memory, implicit memory, and linguistic ability are typically preserved [5]. Memory impairment tends to be both more prevalent and more severe among patients with progressive forms of MS compared to those with relapsing–remitting MS (RRMS). This discrepancy persists even after a decade of the disease's progression [4]. These deficits can be attributed, at least in part, to specific damage in the hippocampus, such as demyelinating lesions and/or diffuse atrophy [6]. Furthermore, memory issues can be influenced by a range of broader psychosocial factors. These factors may include but are not limited to psychological stress, social support networks, lifestyle choices, and coping strategies adopted by individuals facing the challenges of MS. Thus, the impact of memory impairment in MS is multifaceted, encompassing not only the progression of the disease itself but also the complex interplay of psychosocial dynamics [7].

Neuropathology has consistently revealed extensive demyelination, neuronal damage, and synaptic abnormalities in the hippocampus of patients with MS [8]. Similarly, MRI studies have confirmed these abnormalities in-vivo. Hippocampal lesions are frequently seen in individuals with MS, and hippocampal atrophy is a consistent finding at all disease stages. Moreover, it has been found that hippocampal atrophy correlates with deficits in verbal and visuospatial memory performance, even in the early phases of MS [6]. Assessing hippocampal atrophy through imaging is a promising approach to gain insights into the mechanisms and neuroanatomical basis of MS-related memory impairment, particularly when performed at the onset of the disease.

It is important to note here that the hippocampus is not a uniform brain structure and consists of various subfields with distinct structures and functions. Previous cross-sectional studies have suggested different patterns of hippocampal damage throughout the course of MS, initially affecting the cornu ammonis (CA) region [9]. In a study with 23 RRMS patients, 11 SPMS patients, and 18 healthy controls, early CA1 hippocampal volume loss was seen in RRMS, worsening in SPMS, correlating with declining verbal learning, especially in word-list tasks. The subiculum, linked to CA1, also correlated with word-list learning [9]. Another study of 53 pediatric MS patients showed more CA1 and subiculum atrophy, impacting cognition more than overall atrophy [10]. Longitudinal studies in 56 clinically isolated syndrome (CIS) patients found hippocampal atrophy starting from the dentate gyrus and progressing to CA1, correlating with verbal memory deficits at one year. Despite a small sample, the dentate gyrus remained a significant predictor of CA1 and overall hippocampal volumes after one year [11]. However, most of these studies were conducted at a single center and included a small number of patients, potentially limiting the generalizability of their findings.

Against this background, our work focused on a relatively large cohort of early RRMS patients with the overall aim to gain insights into the relationship between hippocampal atrophy and memory function by (i) assessing whether there is global and regional hippocampal atrophy observable at this early stage of the disease, and (ii) investigating the correlation between hippocampal atrophy and memory performance in patients with varying degrees of impairment.

## Methods

### Population

The INNI initiative has supported the creation of a repository where 3-T MRI, clinical, and neurophysiological data from MS patients and HC are collected from four Italian MS Centers (San Raffaele Scientific Institute, Milan, Center A; University Campania “Luigi Vanvitelli”, Naples, Center B; Sapienza University of Rome, Rome, Center C; and University of Siena, Siena, Center D), with the main goal of improving the application of MRI to identify novel MRI markers in MS [12]. For this study, patients were retrospectively selected from the INNI database, with the following inclusion criteria: diagnosis of RRMS [13]; time from disease onset ≤ 5 years; availability of memory assessment using the Selective Reminding Test (SRT) and the Spatial Recall Test (SPART); volumetric 3D-T1W brain MR images acquired at 3 T at the time of the memory assessment.

A population of age-matched healthy controls (HC) was selected from the INNI database and the freely available datasets of IXI (Brain-development.orghttps://brain-development.org) and Nathan Kline Institute (NKI) / Rockland Sample https://fcon_1000.projects.nitrc.org/indi/pro/nki.html.

### Neuropsychological assessment:

At the time of MRI, episodic memory efficiency was assessed by the following two tests. First, the SRT test to evaluate verbal learning and memory performances (including three sub-scores: SRT-LTS = long-term storage; SRT-CLTR = consistent long-term retrieval; SRT-DR = delay recall). SRT test involves presenting participants with a list of words to memorize and then reminding them of specific words they forget over a maximum of six trials, thus providing insights into an individual's ability to acquire and retain verbal information [14].

Second, the SPART test to assess visual or spatial learning and memory (including its delayed recall: SPART-DR = delay recall) [15]. SPART involves presenting participants with a chessboard with ten checkers arranged in a specific layout for ten seconds. Then, they are asked to recall the layout either immediately or after a delay, thus evaluating the ability to remember and mentally manipulate spatial information, which is crucial for tasks such as navigation and orientation. Both SRT and SPART have been largely used in studies involving MS patients to assess memory deficits associated with the condition [16, 17].

Corrected scores for age, sex, and education according to Italian normative values [18] were standardized using z-scores from healthy subjects for each memory test. These scores were then compared to the reference values of SRT and SPART, which characterize each of the 5 cognitive phenotypes proposed [17]. In the original study, the cognitive phenotypes were not linked to impairment in a single cognitive domain; they were derived from a multimodal analysis that included six cognitive tests simultaneously. Therefore, patients categorized under a phenotype might have concurrent impairments in multiple cognitive domains. For the purpose of our study, we focused on memory functions from the cognitive phenotypes. Patients were classified as mildly impaired (MMI) if their memory test performances resembled the first two phenotypes (preserved cognition and mild verbal-memory/semantic fluency) thus showing a deviation from normative memory test scores of less than one standard deviation; or severely impaired (SMI) if their memory test performances resembled the last three phenotypes (mild multidomain, severe executive/attention, severe multidomain), thus exceeded one standard deviation from normative memory test scores. In details, in order to identify the cognitive phenotype (denoted as ${{\varvec{f}}}^{{\varvec{j}}}$) that best matched each subject, (denoted as${{\varvec{s}}}_{i})$, based on their memory test performance, as assessed by SRT and SPART, we followed the following approach.

1. Using the Italian normative values [18], we calculated for each subject the pair of values $${{\varvec{s}}}_{i}$$=($${zscore}_{SRT, }{zscore}_{SPART})$$.
2. We then constructed 2 vectors, ${{\varvec{Z}}}_{SRT}$ and ${{\varvec{Z}}}_{SPART}$**,** whose i-th element corresponds to ${zscore}_{SRT}$ e ${zscore}_{SPART}$ for the i-th subject, respectively.
3. As the two vectors are not independent, so that their correlation ${\rho }^{2}$ is different from 0 and equal to 0.49 for each phenotype we calculated the probability of the density function using the formula:
  $$f\left(x,y\right)=\frac{1}{2\pi {\sigma }_{x}{\sigma }_{y\sqrt{{(1-\rho )}^{2}}}}{\text{exp}}\left(-\frac{1}{2\left[1-{\rho }^{2}\right]}\left[{\left(\frac{x-{\mu }_{x}}{{\sigma }_{x}}\right)}^{2}-2\rho \left(\frac{x-{\mu }_{x}}{{\sigma }_{x}}\right)\left(\frac{y-{\mu }_{y}}{{\sigma }_{y}}\right)+{\left(\frac{y-{\mu }_{yx}}{{\sigma }_{y}}\right)}^{2}\right]\right)$$

where: (x,y) are the z-scores for the i-th subject $${{\varvec{s}}}_{i}$$=($${zscore}_{SRT, }{zscore}_{SPART})$$, $${(\mu }_{x}$$, $${\sigma }_{x})$$ e $${(\mu }_{y}$$, $${\sigma }_{y})$$ respectively are the mean and standard deviation of SRT and SPART and related to a specific phenotype; $${\rho }^{2}$$ is the correlation between $${{\varvec{Z}}}_{SRT}$$ and $${{\varvec{Z}}}_{SPART}$$.

### MRI acquisition and analysis

All MRI scans were collected from the INNI repository between 2008 and 2017. Using 3.0 T scanners, 3DT1-weighted scans were acquired in each center and images quality checked using the protocol previously reported [19]. All the 3DT1-weighted images of MS subjects were lesion-filled [20] and inhomogeneity corrected with N4 [21]. All 3DT1-weighted images of HCs were inhomogeneity corrected with N4, following the same procedure.

**Fig. 1:**
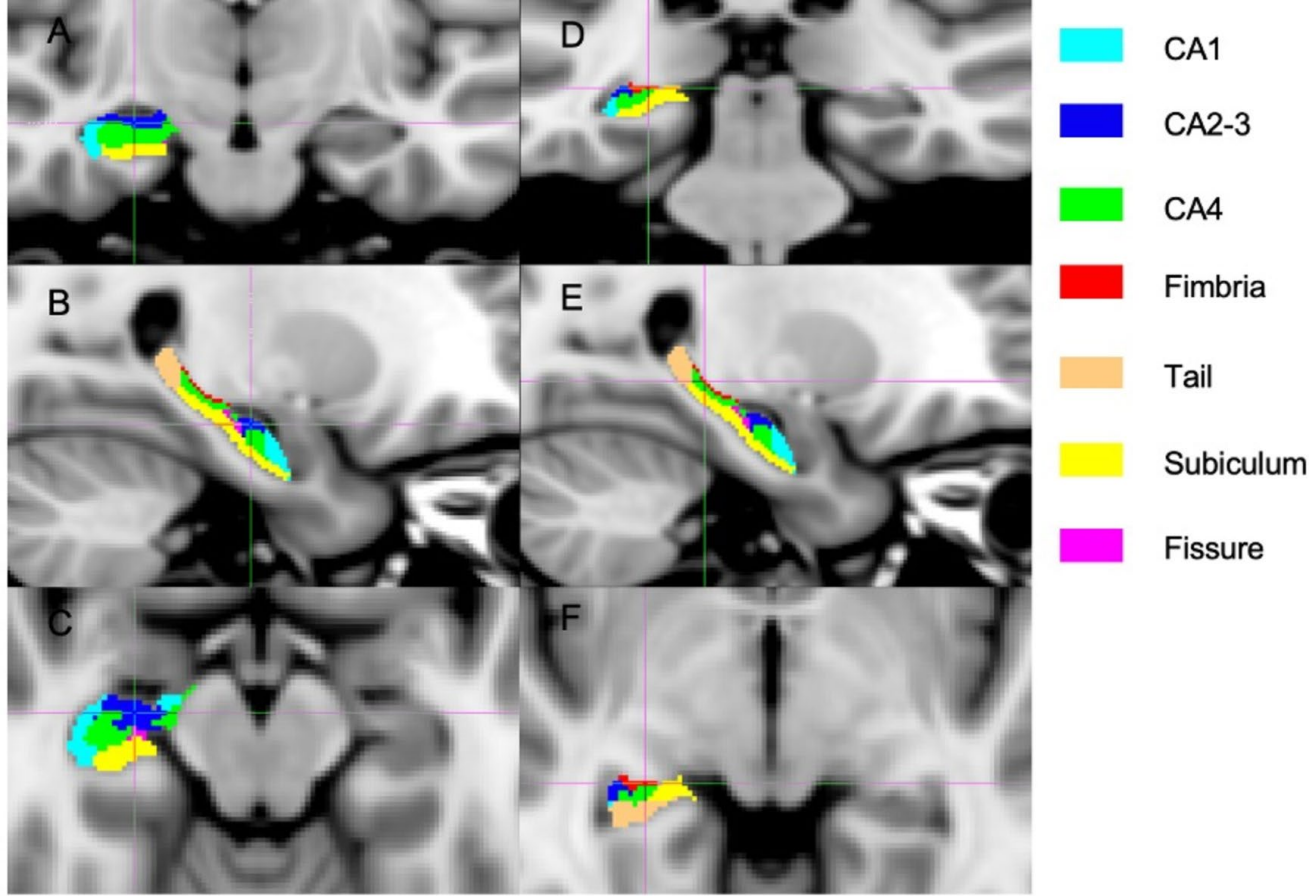
Segmentation of the seven hippocampal subfields. The seven hippocampal subfields automatically segmented using Freesurfer are shown on the anterior (**a**, **b**, **c**) and posterior view (**d**, **e**, **f**), in the coronal (**a**, **d**), sagittal (**b**, **e**), and axial (**c**, **f**) planes

The MRI analysis was divided into the following three steps:

1. Hippocampal volumes generation: A semi-automated approach was used to obtain left and right hippocampal masks. First, on all the 3DT1-weighted images, that were lesion-filled in the MS patients, an initial mask was obtained using FIRST [22]. Then, two independent raters manually corrected each hippocampal mask following the EADC-ADNI [23] harmonized protocol to obtain hippocampal binarized masks. Finally, the volumes of left, right, and total hippocampi were calculated as the sum of all the outlined voxels for each mask, multiplied by the dimension of the voxel.
2. Voxel-based analysis: First, we created a symmetrical template within each group by merging the non-linearly registered isotropic 3D T1-weighted images of 50 individuals with MS and 50 HC into the MNI standard space. This template creation process followed a previously outlined method [24], with minor adjustments, particularly in selecting subjects for each group to ensure that brain volumes closely matched percentiles from the 2nd to the 100th. The goal of this procedure was to depict the extent of atrophy across the entire dataset, utilizing T1-weighted refilled images to counteract biases stemming from lesion presence.
  Next, we non-linearly registered the 3D T1-weighted images of all subjects in the study onto the previously generated within-group template. These images were then combined to form a 4D image containing all the registered hippocampal masks. Additionally, to qualitatively assess the extent and location of significant voxels for each voxel-based analysis, we obtained the subfields of the template using FreeSurfer.
3. Hippocampal subfields segmentation: For each subject, hippocampal subfield masks (i.e., CA1, CA2-3, CA4, fimbria, tail, subiculum and fissure) were automatically segmented using Freesurfer (Fig. 1). These masks were corrected excluding all the voxels not included in the manually edited masks of the hippocampus. For each subfield, hippocampal mask volume was derived by multiplying the number of voxels by the dimension of the voxel.

### Statistical analysis

Analyses were performed with the statistical toolbox of Matlab software. Statistical significance was considered when p-values were < 0.05. Analyses were adjusted for age, sex, and scanner.

Generalised linear models (GLM) were performed to compare volumes of the total, left, and right hippocampi between early RRMS and HC, where “subject group” was the dependent variable and volumes, age and sex the explanatory variables. A Spearman correlation between hippocampal volumes (i.e., total, left, and right) and memory tests (i.e., SRT and SPART) was also performed. The same analyses were used to compare MMI-MS and SMI-MS subgroups and to assess the correlation between hippocampal volumes and clinical and memory tests within each subgroup.

Voxel-wise differences in hippocampal volumes between early RRMS and HC were also assessed. Then, the brain voxels that were significantly different between the two groups were selected to evaluate the voxel-wise correlation with memory tests. Voxel-wise analyses were performed using an unpaired t-test within the framework of the GLM, as implemented in the randomise software [25], using 5000 permutations. Threshold-Free Cluster Enhancement (TFCE) method for multiple comparisons was employed. This method assigns a p-value to each voxel by enhancing areas of signal that exhibit some spatial contiguity without relying on hard-threshold-based clustering. Thus “Cluster-like structures are enhanced but the image remains fundamentally voxelwise” [26] (https://fsl.fmrib.ox.ac.uk/fsl/fslwiki/Randomise/UserGuide). Voxels were considered significant when p < 0.05 (TFCE cluster corrected).

Finally, the same analyses previously performed for whole, left and right hippocampal volumes were repeated using only those subfields that contained atrophic voxels after the first voxel-wise analysis. Then, the Spearman correlations with memory tests were repeated using only those subfields containing significant voxels after the voxel-wise correlation analysis. The same analyses were performed to compare MMI-MS and SMI-MS groups and to assess the correlation between subfields volumes and clinical and memory tests within each subgroup.

## Results

### Participant characteristics

The demographic and clinical characteristics of the participants are summarized in Table 1. A total of 219 early RRMS patients (150 F, mean [± SD] age: 35 [± 10] years, median [range] disease duration: 2 [0–5] years, median [range] EDSS: 1.5 [0–6]) and 246 HC (133 F, mean [± SD] age: 34 [± 9] years) were included in the study. According to their memory test performances, 109 MS patients were defined as MMI-MS and 110 as SMI-MS (Table 1).

**Table 1:** Demographic, clinical and imaging characteristics of patients with early relapsing–remitting multiple sclerosis (RRMS), stratified in mildly memory impaired (MMI-MS) and severely memory impaired (SMI-MS), and healthy controls (HC)

	Early RRMS (N. 219)	HC (N. 246)	p-value	MMI-MS (N. 109)	SMI-MS (N. 110)	p-value
Age, years (mean ± sd)	35 ± 10	34 ± 9	0.31	31 ± 7	38 ± 11	**0.01**
Sex (F/M)	150/69	133/113	**0.015**	64/45	86/24	**0.002**
Disease duration, years (median, range)	2, 0–5	NA	NA	2, 0–5	2, 0–5	0.9
Education, years (median, range)	13, 5–20	NA	NA	14.91 ± 3	13.25 ± 3.2	**0.001**
EDSS (median, range)	1.5, 0–6	NA	NA	1.5, 0–6	1.5, 0–6	0.5
SRT-LTS (mean z-score ± sd)	0.43 ± 1.7	NA	NA	1.19 ± 1.1	− 0.3 ± 1.9	** < 0.001**
SRT-CLTR (mean z-score ± sd)	0.31 ± 1.7	NA	NA	1.04 ± 1.1	− 0.4 ± 1.8	** < 0.001**
SRT-DR (mean z-score ± sd)	0.4 ± 1.6	NA	NA	1.06 ± 0.9	− 0.25 ± 1.9	** < 0.001**
SPART (mean z-score ± sd)	0.14 ± 1.7	NA	NA	0.89 ± 1	− 0.6 ± 2	** < 0.001**
SPART-DR (mean z-score ± sd)	0.09 ± 1.46	NA	NA	0.73 ± 0.9	− 0.55 ± 1.6	** < 0.001**
Number (%) of patients on: Platform DMT^§^	113 (52%)	NA	NA	59 (54%)	54 (49%)	0.45
Number (%) of patients on: High-efficacy DMT^§^	33 (15%)	NA	NA	18 (17%)	15 (14%)	
Total hippocampal volume, mm^3^ (mean ± sd)	7976 ± 856	8315 ± 886	** < 0.001**	8006 ± 802	7969 ± 824	0.34*
Right hippocampal volume, mm^3^ (mean ± sd)	4059 ± 441	4237 ± 468	** < 0.001**	4075 ± 455	4055 ± 423	0.24*
Left hippocampal volume, mm^3^ (mean ± sd)	3916 ± 440	4077 ± 442	** < 0.001**	3931 ± 457	3913 ± 430	0.49*
Scanner vendor	102 Philips	106 Philips		50 Philips	52 Philips	
	47 Siemens	103 Siemens		29 Siemens	18 Siemens	
	70 GE	37 GE		30 GE	40 GE	

Bold values indicate significant differences

*DMT*  disease modifying treatment, *EDSS*  expanded disability status scale, *SRT*  selective reminding test, *SRT-LTS*  long-term storage, *SRT-CLTR*  consistent long-term retrieval, *SRT-DR*  delay recall, *SPART*  spatial recall Test, *SPART-DR*  delay recall

^*^Corrected for age, sex and scanner

^§^Platform DMT included: interferon, glatiramer acetate, teriflunomide, dimethylfumarate. High-efficacy DMT included: cladribine, fingolimod, natalizumab, rituximab

### Differences in hippocampal volumes and correlations with memory tests

Early RRMS showed lower volumes in the whole, right and left (all p < 0.001) hippocampi in comparison to HC (all p < 0.001) (Table 1), and worse scores in memory tests were associated with lower hippocampal volumes (Table 2).

**Table 2:** Correlations between hippocampal volumes and memory test in early relapsing remitting multiple sclerosis (RRMS) and subgroups assessed using Spearman test

	Whole hippocampus			Right hippocampus			Left hippocampus		
	RRMS	MMI-MS	SMI-MS	RRMS	MMI-MS	SMI-MS	RRMS	MMI-MS	SMI-MS
SRT-LTS r (p-value)	**0.194 (0.004)**	**0.249 (0.009)**	0.176 (0.065)	**0.187 (0.006)**	**0.257 (0.007)**	0.153 (0.113)	**0.191 (0.005)**	**0.230 (0.017)**	**0.190 (0.049)**
SRT-CLTR r (p-value)	**0.196 (0.004)**	**0.257 (0.007)**	0.158 (0.099)	**0.199 (0.003)**	**0.253 (0.008)**	0.169 (0.078)	**0.180 (0.008)**	**0.250 (0.009)**	0.137 (0.156)
SRT-DR r (p-value)	**0.207 (0.002)**	**0.347 (< 0.001)**	0.133 (0.169)	**0.210 (0.002)**	**0.365 (< 0.001)**	0.121 (0.210)	**0.193 (0.004)**	**0.313 (0.001)**	0.135 (0.162)
SPART r (p-value)	0.077 (0.258)	**0.344 (< 0.001)**	− 0.073 (0.452)	0.081 (0.233)	**0.344 (< 0.001)**	− 0.068 (0.485)	0.068 (0.316)	**0.328 (0.001)**	− 0.073 (0.451)
SPART-DR r (p-value)	0.115 (0.091)	**0.251 (0.009)**	0.040 (0.683)	0.129 (0.058)	**0.242 (0.012)**	0.067 (0.470)	0.095 (0.165)	**0.248 (0.010)**	0.007 (0.942)

Bold values indicate significant differences

*MMI*  mildly memory impaired, *SMI*  severely memory impaired, *SRT*  Selective Reminding Test, *SRT-LTS*  long-term storage, *SRT-CLTR*  consistent long-term retrieval, *SRT-DR*  delay recall, *SPART*  Spatial Recall Test, *SPART-DR*  delay recall

No differences in hippocampal volumes were found between MMI-MS and SMI-MS patient groups (Table 1). In the MMI-MS group, lower hippocampal volumes correlated with worse scores at all memory tests (r ranging from 0.19 to 0.36, all p ≤ 0.01). In the SMI-MS group, only a weak correlation was found between lower left hippocampal volume and worse SRT-LTS (r = 0.190, p = 0.049) (Table 2).

### Voxel-based analysis

**Fig. 2:**
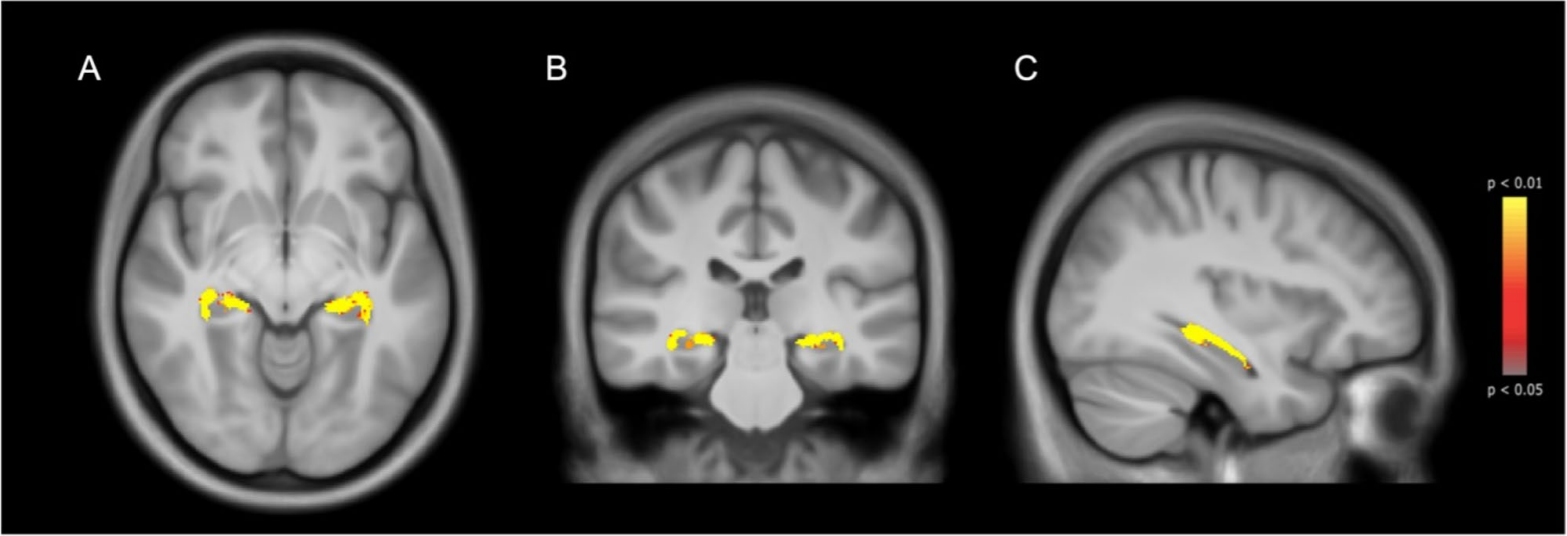
Differences in hippocampal volumes between early relapsing–remitting multiple sclerosis (RRMS) and healthy controls (HC). The figure shows the hippocampal regions with higher atrophy in early RRMS compared to HC in axial (**a**), coronal (**b**) and sagittal (**c**) view. Atrophied voxels are shown in a colour scale from yellow to red, from the most to the less significant, respectively

Results of the VBM analysis showed 5601 atrophic voxels in the group of early RRMS when compared to HC (Right: 2836, Left: 2765) (Fig. 2). When calculating for each segmented subfield the percentage of significant atrophic voxels relative to that subfield, we found that all subfields were involved in a percentage ranging from 79% of the total voxels in the left CA2-3 to 21% in the right tail (Supplementary Table 1). A higher percentage of atrophic voxels in the CA1, CA2-3, and the tail bilaterally correlated with worse cognitive performances at all memory tests, while in CA4 and fimbria correlations were found only with SPART and SPART-recall. No correlations between atrophic voxels and memory tests were found for subiculum and fissure (Supplementary Table 2).

### Differences in hippocampal subfields volumes and correlations with memory tests

Early RRMS showed lower volumes in all hippocampal subfields compared to HC, while no differences were found between MMI-MS and SMI-MS groups (Table 3).

**Table 3:** Differences in averaged volumes of each hippocampal subfield between early relapsing remitting multiple sclerosis (RRMS) and healthy controls (HC)

Subfields	Early RRMS volume, mm^3^ mean (SD)	HC volume, mm^3^ mean (SD)	p-value
*Right hippocampus*			
Subiculum	883.72 (131.9)	976.27 (140.3)	** < 0.001**
CA1	844.44 (137.5)	883.53 (140)	**0.001**
CA2-3	280.44 (58)	294.55 (56.6)	**0.005**
CA4	788.47 (97.5)	821.49 (115.2)	** < 0.001**
Fimbria	39.91 (25.1)	45.24 (24.6)	**0.034**
Fissure	97.27 (38.1)	101.28 (43)	0.113
Tail	513.66 (84.4)	535.92 (107.4)	**0.008**
*Left hippocampus*			
Subiculum	907.24 (140.4)	993.90 (132.8)	** < 0.001**
CA1	797.25 (139.8)	823.88 (139.4)	**0.034**
CA2-3	250.61 (53)	268.66 (54.4)	** < 0.001**
CA4	740.25 (90.7)	786.87 (112.8)	** < 0.001**
Fimbria	42.81(26.8)	50.54 (24.2)	** < 0.001**
Fissure	91.1 (37.7)	95.54 (44.8)	**0.01**
Tail	499.9 (102.2)	513.1 (99.3)	0.06

CA  *cornu ammonis*

**Fig. 3:**
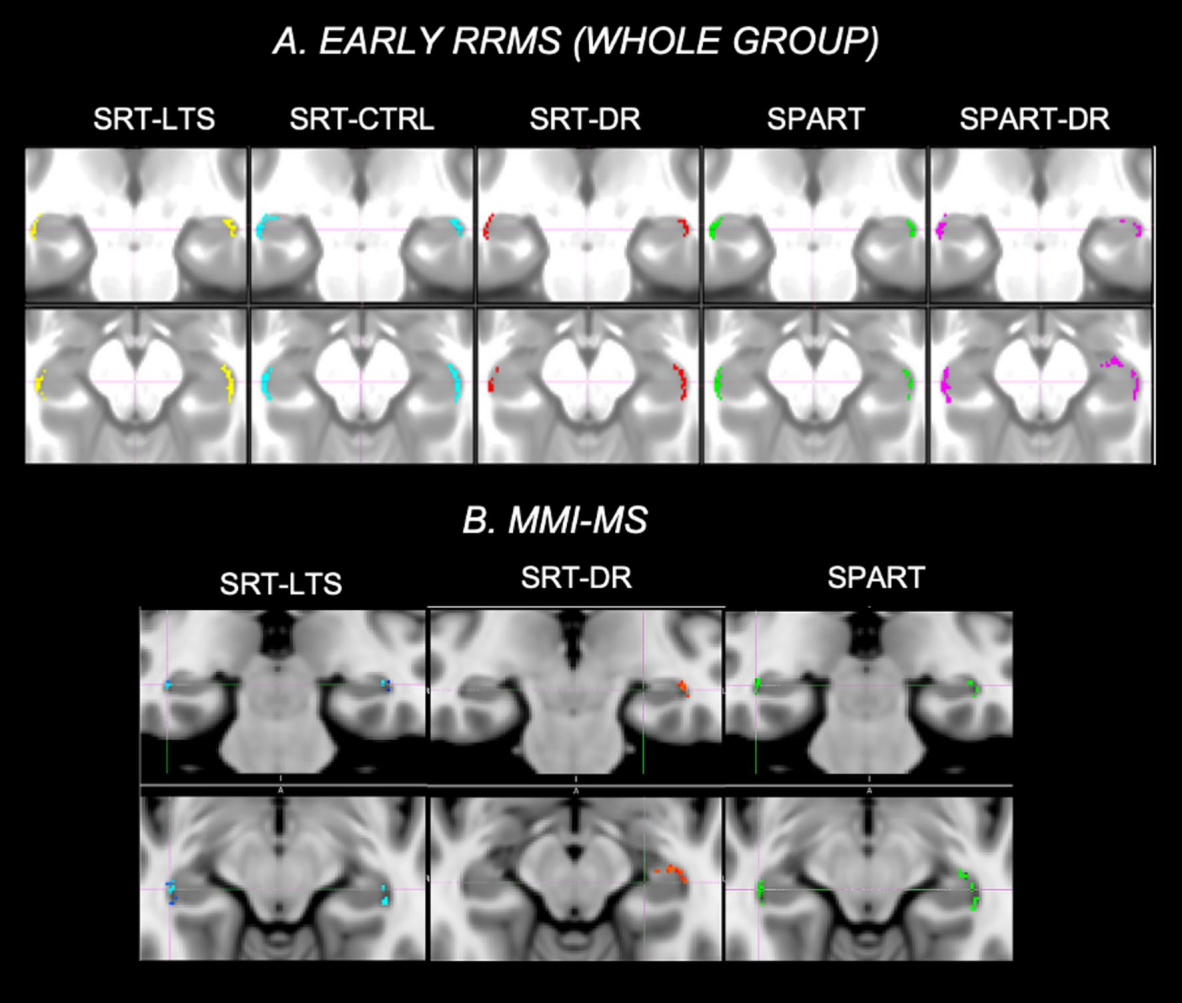
Correlations between hippocampal subfields and memory tests in the whole group of early relapsing–remitting multiple sclerosis (RRMS) and in the mildly memory impaired (MMI)-MS subgroup. The figure shows highlighted in different colors the voxels significantly correlating with memory tests in early RRMS (**a**) and in the MMI-MS subgroup (**b**). In Early MS, reduced subfield volumes correlated with decrease in memory performances, with the closest correlations with right CA1 volume, which was the highest in the MMI-MS subgroup

In the MMI-MS group, however, lower subfield volumes correlated with worse memory performances and this correlation was particularly close between the right CA1 volume and some cognitive tests (SRT-recall: r = 0.38; SPART: r = 0.34). In the SMI-MS group, only weaker correlations were found between subfield volumes and memory tests (Table 4, Fig. 3).

**Table 4:** Correlations between hippocampal subfield volumes and memory tests in mildly memory impaired (MMI-MS) and severely memory impaired (SMI-MS) patients

	MMI-MS*					SMI-MS**				
	SRT-LTS	SRT-CLTR	SRT-DR	SPART	SPART-DR	SRT-LTS	SRT-CLTR	SRT-DR	SPART	SPART-DR
*Right hippocampus*										
Subiculum	0.16	**0.20**	**0.26**	**0.23**	0.15	0.15	0.06	0.10	**− 0.21**	− 0.13
CA1	**0.31**	**0.26**	**0.38**	**0.34**	**0.29**	0.05	0.07	0.01	0.12	**0.24**
CA2-3	0.10	0.13	0.15	0.17	0.08	0.10	0.14	0.07	0.07	0.14
CA4	0.14	0.18	**0.28**	**0.28**	0.14	**0.24**	**0.27**	**0.20**	− 0.15	− 0.02
Fimbria	0.00	− 0.04	− 0.06	− 0.05	− 0.03	0.03	− 0.06	0.01	− 0.07	− 0.04
Fissure	**0.22**	**0.27**	**0.27**	0.11	0.00	0.05	0.08	0.03	**− 0.21**	**− 0.22**
Tail	**0.23**	**0.19**	**0.28**	**0.28**	**0.27**	0.08	0.08	0.08	− 0.11	− 0.02
*Left hippocampus*										
Subiculum	0.12	0.09	**0.22**	**0.20**	0.15	0.04	− 0.03	0.04	− 0.16	− 0.11
CA1	0.17	**0.22**	**0.25**	**0.33**	**0.22**	0.21	0.16	0.14	0.12	**0.19**
CA2-3	− 0.02	0.10	0.05	0.07	0.01	**0.22**	0.18	0.13	− 0.03	− 0.01
CA4	0.06	0.08	0.09	**0.21**	0.10	**0.22**	**0.24**	**0.19**	**− 0.21**	− 0.13
Fimbria	0.10	0.01	0.07	0.09	0.12	− 0.06	− 0.12	− 0.03	− 0.14	− 0.17
Fissure	0.07	0.05	0.13	0.02	0.11	0.09	0.08	0.02	− 0.17	− 0.14
Tail	**0.24**	**0.20**	**0.25**	**0.23**	**0.29**	0.15	0.08	0.02	**− 0.20**	− 0.09

Correlation coefficients are listed in the table and significant results are in bold

CA  *cornu ammonis*

^*^All significant correlations were p ≤ 0.01

^**^All significant correlations 0.05 < p < 0.01

## Discussion

In this multicenter study, we found that hippocampal atrophy is a distinctive feature of the early stages of MS and might represent a reliable marker of memory dysfunction, particularly in patients with mild impairment. When we performed an analysis of hippocampal subfields to explore the different vulnerability of the hippocampal structures to MS-related pathogenic mechanisms, we found a relevant role of the CA adding to previous studies on the specific susceptibility of this region to damage and its subsequent impact on memory function.

We observed significant hippocampal atrophy in both the right and left hippocampi of MS patients when compared to HC, indicating widespread tissue damage in the hippocampus during the early stages of the disease. These findings align with recent MRI studies that have also reported a decrease in hippocampal volume not only at the initial demyelinating event but also over time, suggesting ongoing structural changes in the hippocampus throughout the course of the disease [11, 27]. Importantly, our study extends previous research by demonstrating that, despite the presence of similar hippocampal atrophy in patients with MMI-MS and SMI-MS during the early stages of the disease, only the MMI-MS subgroup exhibited a correlation between hippocampal volumes and memory performance. This might be due to other mechanisms that contribute to memory impairment from the earliest stages of the disease, which cannot be solely explained by reduced hippocampal volume. In line with this hypothesis, a recent study has identified different cognitive phenotypes in MS and found a selective and clinically meaningful hippocampal atrophy in the mildly impaired group, while a widespread brain atrophy and high lesion load was detected in severely impaired patients [17].

An additional interpretation of our findings may lie in the different expression of compensatory mechanisms between the two groups, potentially attributed to variations in brain plasticity and widespread neuronal activation. This differential expression of compensatory mechanisms may account for the favorable clinical outcome observed in patients with MMI-MS. This hypothesis is supported by previous studies that have demonstrated compensatory mechanisms, such as increased structural and functional connectivity, in cognitively preserved MS patients during the early stages of the disease, particularly in those with mild structural abnormalities [27, 28]. These compensatory mechanisms appear to be most effective within the first two years of MS with an efficacy that diminishes as the disease progresses and finally reaches a plateau [29]. Thus, it can be speculated that in patients with MMI-MS, even during these early stages, a compensatory functional reorganization of damaged hippocampal tissue may occur, mitigating the loss of hippocampal volume and temporarily preventing the onset of more severe memory impairment. Future studies assessing the relationship between structural and functional connectivity in MMI-MS and SMI-MS patients would further improve our understanding in this area and provide valuable insights into the compensatory processes occurring in the hippocampus. In addition, the reduced hippocampal volume loss in MMI-MS patients might also be associated with those with mild impairments effectively utilizing cognitive compensatory strategies, or potentially having received some form of intervention.

Consistent with previous studies examining pathology and imaging, our research revealed different vulnerabilities of hippocampal subfields during the earliest stages of MS, which may impact memory performance. While atrophic voxels were detected in all subfields, the CA region appeared to be the most severely affected, whereas the hippocampal tail showed a relatively preserved structure. The CA region is commonly identified as the most vulnerable area in various neurological disorders, and its heightened vulnerability to damage in MS is well-documented [9, 11]. This susceptibility can be attributed to factors such as increased exposure to hypoxic damage and glutamate-mediated excitotoxicity [30, 31]. Additionally, recent studies have demonstrated an increased permeability of the blood–brain barrier (BBB) during the normal aging process, with initial changes occurring in the hippocampus, particularly in the CA region [30, 31]. This breakdown of the BBB has also been observed in patients with mild cognitive impairment but not in young and cognitively preserved MS patients, potentially due to limited sample sizes [30]. On the other hand, the hippocampal tail is known for its robust connectivity and high neuronal plasticity, playing a crucial role in long-term potentiation. A recent study investigating hippocampal subfields reported larger volumes in the tail region in MS patients carrying the brain-derived neurotrophic factor (BDNF) Val66Met polymorphism, which is associated with a protective effect in MS. This finding further emphasizes the potential for functional compensation within this specific subfield of the hippocampus [32]. When examining the correlations between hippocampal volumes and memory performances, these correlations remained numerous and significant in the MMI-MS subgroup. In contrast, only weaker correlations were observed in the SMI-MS subgroup. This highlights the distinct vulnerability of hippocampal structures to MS-related pathogenic mechanisms at different stages of MS.

From a clinical perspective, understanding the varying vulnerability of different parts of the hippocampus in the early stages of MS could have practical implications for evaluating the effectiveness of treatment approaches, especially when certain psychotherapy methods are available to enhance cognition [33]. Apart from being prone to damage, the hippocampus also shows heightened synaptic plasticity and potential for neurogenesis [34], which are thought to respond to exercise training effects [35]. Recent research suggests using changes in hippocampal volume as an outcome measure in clinical trials. For example, in a recent trial involving MS patients with learning and memory issues, treadmill walking exercise was linked to preserved hippocampal volume, whereas the control group exhibited atrophy [36]. Identifying a specific hippocampal subfield that is vulnerable to damage early on could aid in both selecting patients for early intervention and assessing the early effects of treatment. Therefore, the involvement of the CA region in early MS patients implies that targeting this specific area could be promising for cognitive rehabilitation interventions aimed at enhancing memory.

From a technical standpoint, the MRI analysis procedure used here has shown to be feasible and the reported results indicate that hippocampal volume can serve as a reliable imaging biomarker for assessing memory impairment, even in a multicenter setting such as a clinical trial. One of the challenges encountered when evaluating hippocampal atrophy in MS is the lack of standardized methods and tools for analysis. To address this issue, we utilized a semiautomated method following the protocol recommended by EACD-ADNI [23], which strikes a balance between accuracy and reliability/reproducibility. Additionally, we incorporated a lesion-filling procedure to address potential biases and challenges associated with hippocampal assessment in the MS group. This approach allowed us to mitigate subtle changes at the hippocampus interface with other structures, which could have introduced bias during the manual refinement process. By implementing these methods, we were able to enhance the accuracy and robustness of our analysis [37, 38].

This study is not without limitations. First, the cross-sectional design of the study did not allow for an investigation of volume changes over time (i.e., from the disease onset to the evaluation). Conducting further longitudinal analyses would be valuable in identifying patterns of atrophy at different stages of the disease. Additionally, it is important to recognize that the impact of other MS symptoms, such as depression, as well as other measures of neuroinflammation, which have been demonstrated to potentially influence hippocampal damage in MS, were not specifically examined in this study [39, 40]. Another limitation of this study is the lack of available information regarding whether any of the recruited MS patients received cognitive rehabilitation or psychotherapy before the study, which could potentially influence the interpretation of results due to the neuroplastic changes induced by such interventions. Lastly, the analysis conducted on hippocampal subfields might be subject to a reduction in statistical power. Consequently, any interpretations or speculations derived from these subgroup analyses should be approached with caution.

In conclusion, our study provides valuable insights into the involvement of the hippocampus in patients with early RRMS. The relationship between atrophy in specific hippocampal regions, particularly the CA, and impairment of verbal and spatial processing performance points out the relevance of memory-related processes from the early phases of the disease. Moreover, the association between hippocampal atrophy and memory deterioration in the MMI-MS group only, argues for the presence of efficient but saturable compensatory mechanisms even during the early stages of disease. Future studies are needed to elucidate the pathogenetic mechanisms underlying early hippocampal atrophy in RRMS, enhance our understanding of neuronal loss in this brain region, explore its association with inflammation, and evaluate the potential impact of current treatments on this process.

### Supplementary Information

Below is the link to the electronic supplementary material.

Supplementary file1 (DOCX 17 KB)

## Data Availability

Anonymized data not published within this article will be made available by request from any qualified investigator.
