# Supplementary material for: Regional hippocampal atrophy reflects memory impairment in patients with early relapsing remitting multiple sclerosis
Source: J Neurol. 2024 May 14;271(8):4897–908. doi: 10.1007/s00415-024-12290-8 (PMC11319433; doi:10.1007/s00415-024-12290-8)
Supplement: Supplementary file 1 — Supplementary file1 (DOCX 17 KB) [file 415_2024_12290_MOESM1_ESM.docx]

**Online Supplementary materials:**

**Supplementary Table 1:** Percentage of atrophic voxels within each hippocampal subfield in the whole early relapsing-remitting multiple sclerosis group.

| **Hippocampal Subfields** | **Side** | **Percentage (%) of atrophic subfield** |
| --- | --- | --- |
| Subiculum | Right | 30.47 |
| CA1 | Right | 33.42 |
| CA2-3 | Right | 69.48 |
| CA4 | Right | 38.79 |
| Fimbria | Right | 22.52 |
| Fissure | Right | 64.71 |
| Tail | Right | 21.27 |
| Subiculum | Left | 30.23 |
| CA1 | Left | 25.53 |
| CA2-3 | Left | 79.07 |
| CA4 | Left | 40.18 |
| Fimbria | Left | 70.75 |
| Fissure | Left | 87.50 |
| Tail | Left | 26.25 |

*Abbreviations: CA=cornu ammonis*

**Supplementary Table 2:** Percentage of atrophic voxels correlating with memory tests in the whole early relapsing-remitting multiple sclerosis group.

|  | **Memory tests** | | | | |
| --- | --- | --- | --- | --- | --- |
|  | **SRT-LTS** | **SRT-CLTR** | **SRT-DR** | **SPART** | **SPART-DR** |
| **Right hippocampus** | | | | | |
| **Subiculum** | 0 | 0 | 0 | 0 | 0 |
| **CA1** | 3.722 | 6.345 | 2.284 | 3.891 | 5.414 |
| **CA2-3** | 0.744 | 5.21 | 0.744 | 1.736 | 5.955 |
| **CA4** | 0 | 0 | 0 | 0 | 0 |
| **Fimbria** | 0 | 0 | 0 | 0.9 | 1.801 |
| **Fissure** | 0 | 0 | 0 | 0 | 0 |
| **Tail** | 0.693 | 1.965 | 1.04 | 0.462 | 1.849 |
| **Left hippocampus** | | | | | |
| **Subiculum** | 0 | 0 | 0 | 0 | 0 |
| **CA1** | 10.512 | 9.187 | 5.918 | 5.653 | 7.42 |
| **CA2-3** | 7.493 | 6.976 | 1.808 | 6.201 | 12.661 |
| **CA4** | 0 | 0 | 0 | 0 | 0.101 |
| **Fimbria** | 0 | 0 | 0 | 0.943 | 1.886 |
| **Fissure** | 0 | 0 | 0 | 0 | 0 |
| **Tail** | 0.909 | 0.681 | 0.227 | 0 | 0 |

*Abbreviations: CA=cornu ammonis, SRT=Selective Reminding Test, SRT-LTS=long-term storage, SRT-CLTR=consistent long-term retrieval, SRT-DR=delay recall, SPART= Spatial Recall Test, SPART-DR=delay recall.*
